# Predictors of Leisure Participation in 6 to 14-Year-Old Children with Cerebral Palsy: Structural Equation Modeling

**Published:** 2020

**Authors:** Shakiba GHAFFARI, Minoo KALANTARI, Mehdi REZAEE, Alireza AKBARZADEH BAGHBAN

**Affiliations:** 1 Physiotherapy Research Center, Department of Occupational Therapy, school of Rehabilitation, Shahid Beheshti University of Medical Sciences, Tehran, Iran; 2 Department of Occupational Therapy, school of Rehabilitation, Shahid Beheshti University of Medical Sciences, Tehran, Iran; 3 Proteomics Research Center, Department of Biostatistics, School of Allied Medical Sciences, Shahid Beheshti University of Medical Sciences, Tehran, Iran

**Keywords:** Participation, Leisure, Cerebral Palsy, Structural Equation Modeling

## Abstract

**Objective:**

The aim of this study was to test a model of child, family and environment and identify factors affecting the intensity of leisure participation by children with cerebral palsy (CP).

**Materials & Methods:**

In this cross-sectional study, 232 children with cerebral palsy (141 boys and 91 girls), aged 6 to 14 years old and their parents were selected from four schools of children with special needs and five rehabilitation centers through the convenience sampling method in Shiraz, Iran. To evaluate the intensity of leisure participation, we used the Persian version of Children’s Assessment of Participation and Enjoyment (CAPE) completed by the participants. Demographic form, Craig Hospital Inventory of Environmental Factors (CHIEF), Strengths and Difficulties Questionnaire (SDQ), Family Environmental Scale (FES), SPARCLE cognitive level and parents' version of Gross Motor Function Classification System, Manual Ability Classification System and Communication Function Classification System were sent to the parents with some necessary explanations. Structural equation modeling was used to test the model hypothesis. SPSS version 18 and AMOS version 16 were used for data analysis.

**Results:**

Comparative fit indexes indicated a moderate to good model fit. The presented model explained 44% of the variance in the intensity of participation. Constructs such as Family Activity Orientation with standardized total effect of 0.31 and path coefficients of P< .05 showed the most significant direct effect on participation, followed by higher gross motor function (-.26), higher manual ability (-.19), communication function (-.17), higher cognitive level (-.16), more siblings in the family (.15) and less emotional-behavioral problems (-.15). Family structures and relationships (.17) and unsupportive environment constructs (-.14) demonstrated an indirect but significant effect (P< .05). The relationship of family education level and income with participation was not significant (P>.05).

**Conclusion:**

The intensity of CP children’s participation is influenced by child, family and environmental factors. Parents' knowledge of recreational activities and their preference to participate in leisure and recreational activities provide children more opportunities to participate. Higher gross motor function, manual ability, and communication function also play an important role in their participation. Family structure means family cohesion, roles organization, and conflicts between family members and encountering physical, attitudinal and structural barriers at home and community indirectly impact children’s participation pattern. To enhance children’s participation, we suggest therapists to support children’s behaviors, family relationships and involvement in community activities and optimize physical function of children with limitations in self-mobility.

## Introduction

Participation has both subjective and objective aspects and it means engagement in life situations (1). Children’s participation is an important and complex issue that changes with social and cultural contexts of each society (2). Leisure includes voluntary activities that children engage in within their free time with internal motivation (3). It is also an important occupational concept and children spend 30% of their time participating in these activities (4). Leisure activities include athletic, skill-based, physical, recreational and self-improvement activities. Participation in these activities improve children’s physical, social and emotional development, relationships and performance in their home, school and community roles (5). However, most children with physical disabilities perform recreational activities in solitude in home-restricted contexts (6).

Most Iranian articles addressed child participation in activities of daily living, while participation in none of the eight occupation areas is superior to the other (3, 7). Although weakness of cerebral palsy students in leisure activities compared to their normal peers has been reported, there are few studies focusing on their leisure and recreational activities (8, 9). 

King et al. in 2006 examined a model of predictors of leisure participation in children with chronic physical disabilities by SEM statistical analysis. The model explained 30% of the variance in the intensity of participation in informal activities requiring little or no planning and 18% of the variance in the intensity of participation in formal activities structured by adults (10). Palisano and Chiarello in 2011 and 2016 separately examined the rate of leisure participation in cerebral palsy students and children under five years of age using the SEM approach. The results of these studies and some others emphasize the importance of individual factors such as higher gross motor function, higher enjoyment, more effective adaptive behaviors, and younger age as well as the role of family environment, family activity orientation and family ecology. Palisano indicated their model explained 32% of the variance in the intensity of participation, while in the study by Chiarello, the model explained 35% and 40% of the variance in the frequency of participation in recreation. Also, in the studies by Palisano and Kolehmainen, the role of environmental barriers in participation was not meaningful due to measurement errors and model misspecification (11-13).

**Figure 1:**
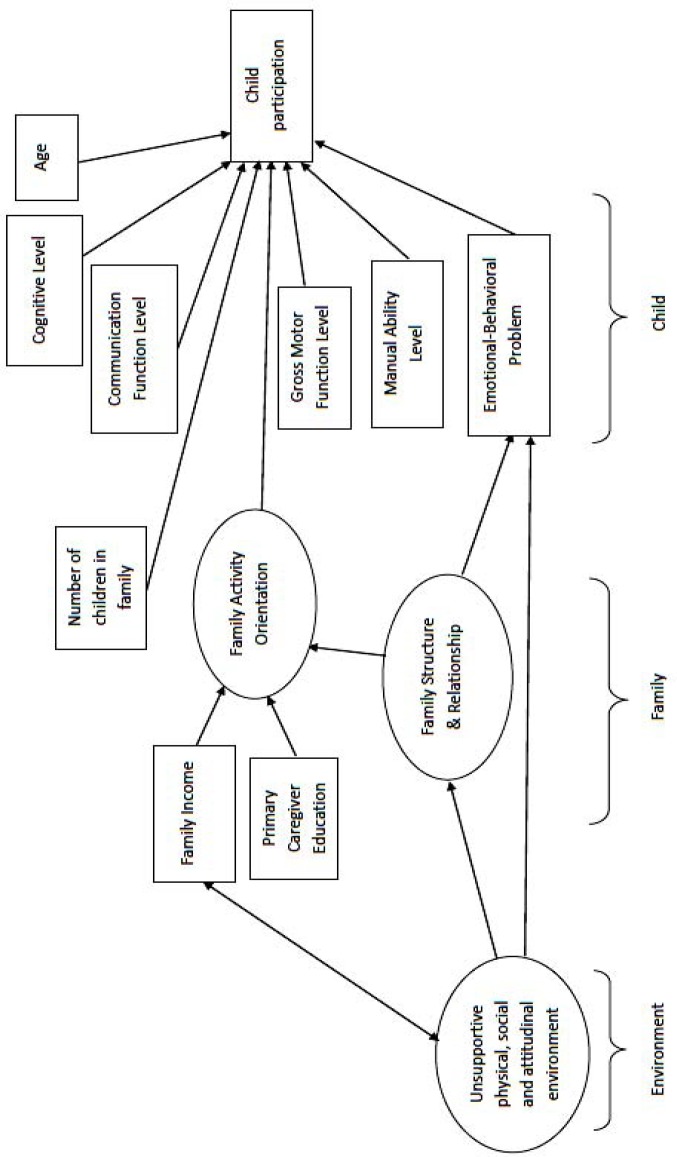
Conceptual and measurement model for the rate of participation in children with cerebral palsy

Nowadays, rehabilitation planning needs more evidence as to the role of variable factors like environment, personal characteristics and family in the rate of cerebral palsy children’s participation in recreational activities. We used structural equation modeling (SEM) as our statistical approach. This method is different from regression and it can examine direct and indirect paths at the same time; it can also take into account latent variables not measured directly in the model (14). The conceptual model of the factors influencing the rate of leisure participation in children with cerebral palsy is presented in Figure 1. It was shaped and linked through discussion in the research team in the process of reviewing the literature and theories. This model indicates that children’s participation is multidimensional and complex and affected by personal, familial and environmental factors. The model includes various paths based on the ecological sequences that constructs can change the participation content and pattern directly or indirectly. We used evidence paths between constructs as hypothesis in our theoretical framework.

**Specific hypothesis in the model:**

**Child-related factors**:

We proposed that: The constructs gross motor function, children manual ability, cognitive level, communication function level, emotional and behavioral problems, and age have a direct effect on participation (4, 12, 15). In fact, children with better physical functioning experience fewer limitations and barriers to participation than those with weak motor ability (16, 17). Cerebral palsy children who do not have behavioral problems and have better communication functions are more likely to be involved in leisure activities along with family and friends (10). We also proposed that high intelligence is related to high rate of leisure participation (15, 18).

**Family-related factors:**

We proposed: Family activity orientation (family’s interest for social, logical and cultural activities and their participation in recreational activities) is related to high rate of children’s participation (10, 12). Primary caregiver education and financial resources are associated with family’s intellectual-cultural orientation and its preferences for recreation and higher child participation (12, 19, 20). Family structures and relationships (i.e., family cohesion, conflicts, activities and roles organization) could change family activity orientation and indirectly promote child participation (10, 12), also it can influence emotional and behavioral problems and indirectly change child’s participation pattern (21). We predicted that the number of siblings and being among peers and friends can increase confidence, motivation and psychosocial support for disabled children and is related to high child participation (22).

**Environment-related factors:**

We proposed that unsupportive physical, attitudinal and social environments, for example, personal assistant services, others’ prejudices, lack of information and architectural barriers are related to family structures and relationships and child’s emotional behaviors (because disability in participation is seen as a person's problem in relation to the environment and facilitating or unsupportive environment affects child’s emotional and behavioral functions), and therefore, higher child participation (10, 21, 23).

Unsupportive environment and income interacted with each other because lower/higher income cause facing less/more environmental barriers (lack of assistant, access to transportation system and accommodation) (24).

Our research objective, which was to test the model of factors influencing the rate of Iranian cerebral palsy children’s participation in leisure activities. Identifying the predictors can help therapists, families, managers, and policymakers to know "which levels of child, family or environment are better to plan on and which factors should be more focused on?"

## Materials & Methods

Participants

In this cross-sectional study, we enrolled 232 cerebral palsy children aged 6 to 14 years old (mean age: 9y 3mo, SD 2.5y) and their parents. Samples were selected from four schools of children with special needs and five rehabilitation centers through the convenience sampling method in Shiraz, Iran, from January 2017 to September 2018. The inclusion criteria were: 1- age range 6 to 14 years, 2- cerebral palsy diagnosis (based on medical record or occupational/physical therapist report), 3- ability to read and write for parents and 4- no history of another neurologic disease. The exclusion criteria included: 1- unwillingness to complete the questionnaires and 2- failure to complete the questionnaires correctly. 

Measurement

We used the Persian version of Children’s Assessment of Participation and Enjoyment (CAPE) for evaluating the rate of leisure participation in children. The CAPE is a 55-item measure of leisure and recreational activities done during the past four months. It is designed for children aged 6 years old and over. The dimension "intensity" ("how often" activities were done) is rated on a seven-point scale from 1, "once in the past four months" to 7, "once a day or more". Item discriminant validity was confirmed by 85% scores for each of the questions in the subscale scores were significant and positive. Alpha Cronbach's coefficient was 0.86 and interclass correlation coefficients were > 0.75 for the overall scale. These scores showed high validity and reliability of the Persian CAPE questionnaire .

The family environmental scale has 90 (true and false) two-way questions. This scale assesses the social climate of the family, the interpersonal relationships of family members and the family's efforts to maintain itself in critical situations. The questionnaire has 10 subscales, and we measured the subscales of cohesion, organization, and conflict for family structure and relationship construct and the subscales of intellectual cultural orientation and family participation in recreation for family activity orientation construct. For the Persian version, the Cronbach's alpha ranged from .56 to .79, and its correlation coefficient spanned from .63 to .83 (25).

We used Craig Hospital Inventory of Environmental Factors (CHIEF) to assess unsupportive physical, social, and attitudinal environments. It is a 25-item scale that quantifies the degree to which aspects of the physical, social, and political environment act as barriers to full participation. The Persian version had good validity and reliability, the Intraclass correlation coefficient was 0.94 and Cronbach's coefficient was 0.86 (26).

*The Strengths and Difficulties Questionnaire (SDQ) evaluates the problems and abilities of children in the age range of 3 to 16 years old using 25 items. The questionnaire consists of self-report, teacher and parent forms, and in this study we used the parent form. The diagnostic reliability and validity was established through the implementation of parent and teacher form in Shahrekord and using the total cut-off point of 19 (* 27 *).*

Gross Motor Function Classification System, Manual Ability Classification System, Communication Function Classification System and SPARCLE cognitive level form were selected for gathering data for child factors (23,28-30). The demographic form included items on age, primary caregiver education and income level in the model. 

**Procedure**

A sample of 300 participants was selected using the inclusion criteria, after obtaining the permission and coordination with the management of the selected rehabilitation centers and schools, informed consent was obtained from parents (they were familiarized with the study objectives). A package of self-administered questionnaires was sent to the families with some essential explanations on how to answer the questions. The parents completed the demographic questionnaire, the Craig Hospital Inventory of Environmental Factors, the Family Environment Scale, the Strengths and Difficulties Questionnaire, Parents version of GMFCS, MACS and CFCS and SPARCLE cognitive level form at home due to time constraints. CAPE was completed by children, and they were allowed to complete the questionnaire with some help from their mother or caregiver, if necessary. 

## Results

Out of 300 questionnaires, 68 were excluded from the study because of lack of full completion, and ultimately only 232 questionnaires were analyzed. Most child participants were at level II and IV GMFCS. Characteristics of the samples are shown in Table 1. Children with cerebral palsy mostly participate in recreational (mean: 34.4, SD: 14.1) and social (mean: 24.6 , SD: 9.9) activities, followed by self-improvement (mean: 13.8, SD: 9.1), physical (mean: 11.3, SD: 7.5) and skill-based (mean: 4.8, SD: .2.6) activities. Most encountered environmental barriers from parents’ views were attitudes and supports (mean: 17.9, SD: 1.01), services and assistants (mean: 8, SD: 5.75), policies (mean: 6.9, SD: 5.31), school (mean: 5.56, SD: 4.88) and physical and structural (mean: 4.5, SD: 3.75). The structural model was tested using the AMOS software program version 16. Skewness and kurtosis of each distribution were acceptable (<2). The structural equation technique is a combination of two analyses: 1. Measurement model (confirmatory factor analysis) and 2. Structural model (path analysis).

**Table 1:** Characteristics of 232 cerebral palsy children and their families

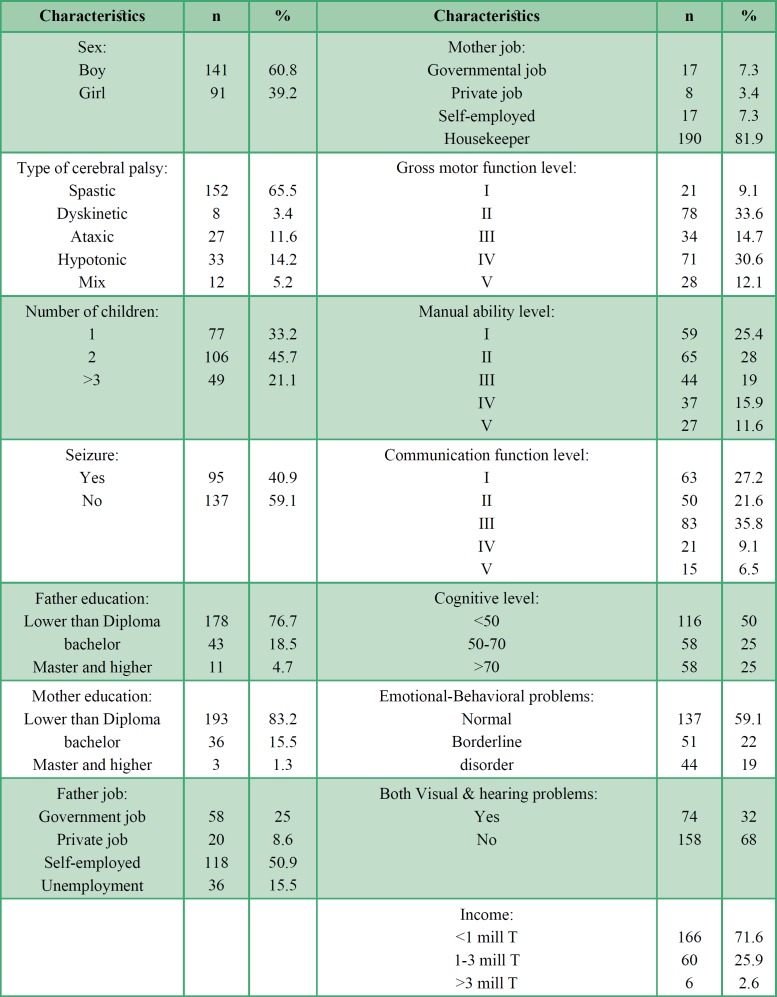

**Measurement model:**

For data analysis and investigating the relationship between factors and determine the role of each variable, first we tested the reliability of the measurement model. Factor analysis was used to examined the reliability and indicators loading of each construct. The loadings of the measured indicators on the factors were of acceptable magnitude (Table2) (32). Then, we determined the correlation among variables (Table 3).

**Table 2:** Indicators and loadings of directly measured variables on constructs

**constructs**	**Source**	**Standardized loading**
**Unsupportive physical, attitudinal and social environment**	Policies (CHIEF) Physical & structural(CHIEF) School & work(CHIEF) Attitudes & supports(CHIEF) Services & assistants (CHIEF)	. 71 . 72 . 77 . 76 . 85
**Family structures and relationships**	Cohesion (FES) Conflicts (FES) Organization (FES)	. 59 -. 65 . 50
**Family activity orientation**	Family intellectual-cultural orientation(FES) Family participation in recreations(FES)	. 54 . 55
**Number of children**	Demographic Questionnaire	
**Cognitive level**	9-item SPARCLE form	
**Gross motor function level**	Parents version of GMFCS	
**Manual ability level**	Parents version of MACS	
**Communication function level**	Parents version of CFCS	
**Emotional-behavioral problems**	Strength & Difficulty Questionnaire	
**Primary caregiver education**	Demographic Questionnaire	
**Income**	Demographic Questionnaire	
**Age**	Demographic Questionnaire	
**Intensity of participation**	CAPE	

*Note. *Empty cells indicate that there was no loading because the construct is measured by a single indicator.

**Table 3:** correlations Among Study variables.

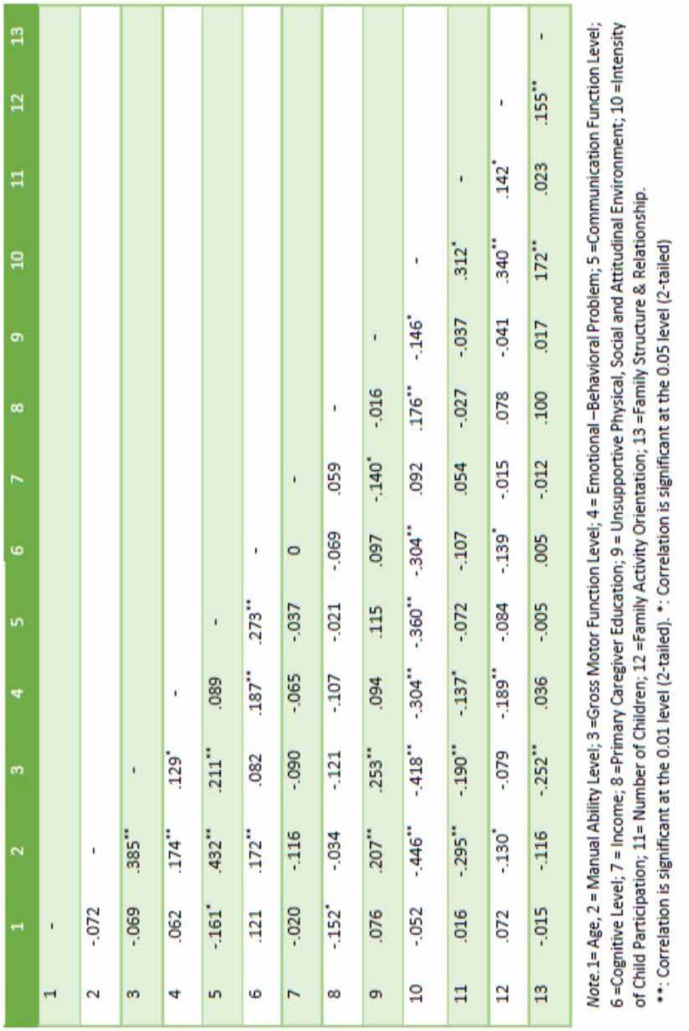

**The structural model:**

SEM explains the relationships between observable and latent constructs, total standardized direct and indirect effects and the variance of the model. Initially, we tested model contained all the paths among factors that were compatible with our theoretically-based model. Three constructs (i.e., age, primary caregiver education, and income) were excluded from the analyses because they reduced the fit of the model (P>.05). To improve model fit, paths from income & education to family activity orientation, income to unsupportive environment and from age to child participation were omitted. The process of model building resulted in two new proposed pathways, all theoretically justifiable: from unsupportive environments to child gross motor function and manual ability level (because environmental barriers limit opportunities for the development of children’s physical function). The final structural equation model presented in Figure 2 showed moderate to good fit indexes. CFI (Comparative Fit Index) = .83, RMSEA (Root Mean Square Error of Approximation) = .07, GFI (Goodness of fit index) = .87, PRATIO=.83 and CMIN/DF=2.5 were at acceptable levels (31). The arrows in the figure indicate statistically significant path coefficients (*p *< .05). Directly measured variables are indicated by squares; latent constructs, consisting of a number of indicators, are indicated by ellipses.

**Figure:**
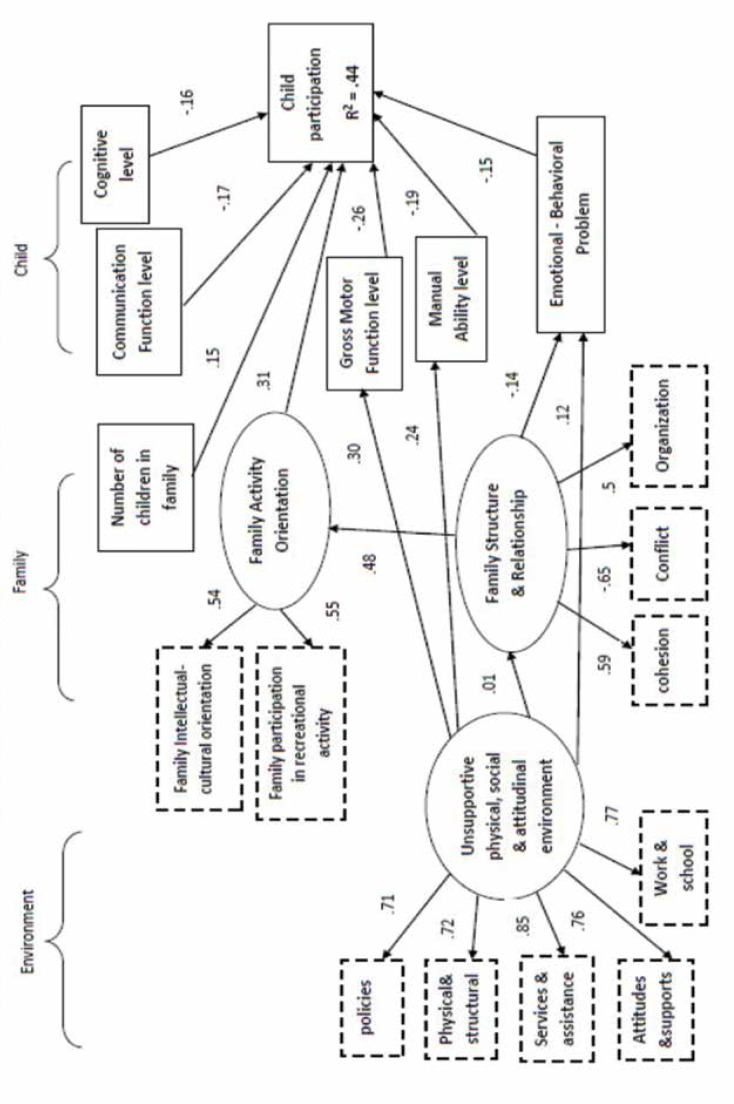


## Discussion

In this study, we used SEM approach to test the theoretical child, family and environment model. The model accounted for 44% of the variance in overall cerebral palsy children’s rate of leisure participation and showed acceptable moderate to good fit indexes.

As expected, there were multiple interrelated determinants of children’s participation. Child factors such as gross motor function, manual ability, communication function had statistically significant roles in child participation, these findings confirmed results in the previous models in King et al. and Palisano et al. studies, which indicated children’s functional abilities and gross motor function had a direct effect on child participation (10, 12). Other studies on leisure participation and daily life activities also indicated the importance of gross motor, manual ability, the communication function and intelligence in child participation (15-18, 33). In two studies, the association of gross motor function and mobility with participation was not significant because of multiple sites sampling and various physical impairments (13, 34).

The main result of this study was the importance of family-related factors, the role of family intellectual-cultural orientation and family preferences to engage in recreation activities. In Family Activity Orientation construct with (.^31^) coefficient just in our study was more than the impact of child factors. In fact, our model supports the hypothesis that family knowledge and information in social activities and engagement in cultural and recreation programs in the community could increase the rate of child participation. These findings are consistent with the results of some studies in the literature (10, 12, 13).

In Chiarello model, family environment and recreational programs in the community were associated with young cerebral palsy children’s participation (11). In some studies, family knowledge of leisure activities and participation in recreations were associated in children with developmental delay and motor impairments (13, 33, 35). It seems positive parental view in choosing leisure activities and encouraging their children could increase their PARTICIPATION. In the review study by Longo et al., personal factors (i.e., intelligence and gross motor function) and environmental factors with 40% and 24% of variance were more significant than family factors (18), this difference might be due to the difference in Iranian society. 

Another noticeable factor was siblings of the children, having brothers and sisters increase motivation, self-confidence and support in disabled children, and therefore, promote their participation. Interpersonal, social and supportive relationships with close friends, teachers and peers are associated with high child participation (10, 13, 21). This study also emphasized that normal behaviors could increase total participation by 15%. Emotional-behavioral problems, for example isolation, hyperactivity and aggression impede the child to participate normally in plays and recreations, because based on behavioral treatment approach, behavioral problems decrease adaptability, coping ability and sense of mastery in activities (10, 15). Palisano et al. and Chiarello et al. indicate the importance of adaptive behaviors using the coping inventory form in cerebral palsy children’s leisure participation(11, 12).

One of the advantages of SEM is the ability to measure indirect pathways. The family factor, that is, Family Structure (family members' cohesion, help, their conflicts and role organization) (β =.17) had a significant indirect effect, which confirmed the results of previous studies (10, 11). Greater family cohesion was related to stronger family orientation toward intellectual-cultural activities and higher family participation in social and recreational activities. 

Family structure is an important factor in child participation process, it can shape the child's behaviors in the future so organizing roles in family, support and helping each other or violence, hard punishment and inhibitory interaction could encourage or impede children’s participation in activities and creativities (36). In Canadian study by Larivière-Bastien et al., youth with cerebral palsy indicated that parental and family support is needed to achieve basic needs (37).

In addition, environmental barriers (β = -.14) play an important role in child participation. In fact, others’ view toward the disabled child, their assistance and help at home or in the society, access to transportation and lack of equipment in terms of policy could hamper children's physical ability and their opportunity to participate. The role of social environment in creating recreational programs was indicated in a former study (11). The unsupportive environment in our model affected gross motor and manual ability by 30% and 25%, respectively. In King's study, environment’s effect was 48% on physical ability, which reminds the importance environmental barriers on physical ability. In other studies, the association of environmental barriers with the participation of children was mentioned, and our findings confirmed their results (38-40).

Despite the elimination of age factor from the final model, in the present study, with increasing age and transition to adolescence, participation in leisure activities, especially recreational activities, decreased because they tend to participate in social activities for their future social role. These findings are in line with findings of other studies in the literature (4, 12, 15, 17, 33, 41). 

**In Conclusion**

Our conceptual model only explains 44% of variances and it means that the model requires additional determinants that could be related to other aspects of family functions, such as collaborative decision making with parents respecting child's needs and interests, membership in groups and the importance of parent-child relationships, and at environment level, for example, community acceptance for people with disabilities and closeness of residence to playgrounds. Overall, regarding children’s leisure participation the most effective services seem to be those based on family function. Family-based services are based on the theory of family system and focus on family strength and abilities and recognition of the needs of children (42). 

According to the importance of family-centered approach in occupational therapy and the importance of family as a means for developing participation and shaping children's social identity, community service providers and occupational therapists should be more active in this area. Enhancing knowledge of activities the child enjoys, family activity orientation and family knowledge of community resources is essential for identifying participation opportunities. Occupational therapists should insist on organizing workshops to identify leisure activities and teaching these activities to families and teachers. The important role of family members' direction and participation in recreational activities can be modified with the rehabilitation team training and advice. In this regard, appropriate activities and programs must be identified and, after careful examination by occupational therapists, their suitability for children’s mental and physical condition should be examined. Also, due to the multitude of environmental barriers in areas of attitudes and support, public policies should extend the services and facilities needed for disabled individuals. Occupational therapists also can resolve these barriers by implementing environmental modifications, activity adaptation and assistive technologies.

## Limitations

one study limitation was non-random and convenience sampling. A large percentage of families due to their child’s low cognitive level filled the CAPE questionnaire by themselves. Moreover, in several studies, the role of family stress and child preferences to participate were mentioned, which was not possible to complete due to the time limit. We did not measure the intelligence score because we did not have the time and financial resources to administer a standardized measure of intelligence.
